# The Placebo Effect in Chronic Thromboembolic Pulmonary Hypertension Trials: A Systematic Review and Meta-Analysis

**DOI:** 10.3390/medsci13020057

**Published:** 2025-05-07

**Authors:** Daniel Caldeira, Daniel Inácio Cazeiro, Rui Plácido, Filipa Ferreira, Rita Calé, Fausto J. Pinto

**Affiliations:** 1 Serviço de Cardiologia, Departamento de Coração e Vasos, Centro Hospital Universitário Lisboa Norte (CHULN), Unidade Local de Saude Santa Maria, Centro Académico de Medicina de Lisboa, 1649-035 Lisboa, Portugalfaustopinto@medicina.ulisboa.pt (F.J.P.); 2 Laboratory of Clinical Pharmacology and Therapeutics, Faculdade de Medicina, Universidade de Lisboa, 1649-028 Lisboa, Portugal; 3 Centro Cardiovascular da Universidade de Lisboa (CCUL@RISE), Faculdade de Medicina, Universidade de Lisboa, 1649-028 Lisboa, Portugal; 4 Centro de Estudos de Medicina Baseada na Evidência (CEMBE), Faculdade de Medicina, Universidade de Lisboa, 1649-028 Lisboa, Portugal; 5 Serviço de Cardiologia, Hospital Garcia de Orta, 2805-267 Almada, Portugal; filipamaferreira@hotmail.com (F.F.); ritacale@hotmail.com (R.C.)

**Keywords:** chronic thromboembolic pulmonary hypertension (CTEPH), placebo effect, clinical trials, systematic review, meta-analysis, 6 min walk test (6MWT), pulmonary vascular resistance (PVR), EQ-5D, natriuretic peptide, pulmonary embolism

## Abstract

**Introduction:** Placebo-controlled studies are crucial in clinical trials, but the placebo effect can vary across conditions. We aimed to assess the placebo effect in chronic thromboembolic pulmonary hypertension (CTEPH) trials. **Methods:** We conducted a systematic review and included randomized placebo-controlled trials investigating CTEPH interventions. Primary outcomes were the pre–post changes in the 6 min walk test (6MWT) and quality of life in the placebo arms. Secondary outcomes included mean pulmonary artery pressure (mPAP), pulmonary vascular resistance (PVR), cardiac index, and NT-proBNP levels. Meta-analyses were performed using random-effects models. **Results:** Seven trials with 270 CTEPH patients in placebo arms were analyzed. The average 6MWT change was not significant (−1.31 m; 95%CI −12.49 to +9.79). Quality of life with EQ-5D was not significantly improved (−0.04; 95%CI −0.10 to +0.02). mPAP, PVR, cardiac index, and NT-proBNP also demonstrated non-significant changes with small magnitudes. **Conclusions:** The placebo effect in CTEPH trials was not statistically significant and had small magnitude but should not discourage the use of placebo-controlled trials where applicable and ethical.

## 1. Introduction

Chronic thromboembolic pulmonary hypertension (CTEPH) is a progressive condition characterized by persistent obstruction of the pulmonary arteries due to unresolved thromboembolic material, leading to increased pulmonary vascular resistance and right heart failure. It represents a unique entity within the spectrum of pulmonary hypertension, differing from pulmonary arterial hypertension in its underlying pathophysiology, treatment approaches, and response to medical therapy [1]. It represents a distinct form of pulmonary hypertension that is potentially curable with pulmonary endarterectomy (PEA); however, a substantial proportion of patients remain inoperable or experience persistent/recurrent pulmonary hypertension post-surgery [2]. Pharmacologic therapy and balloon pulmonary angioplasty (BPA) have emerged as alternative strategies [1], but the role of placebo or sham-controlled trials in this context remains a subject of debate.

The placebo effect, a well-documented phenomenon in clinical trials, can influence subjective outcomes such as exercise capacity and quality of life, potentially confounding treatment efficacy assessment [3,4,5]. Moreover, placebo effects may be magnified in diseases with high symptom variability, such as CTEPH, where functional capacity is influenced by fluctuating hemodynamic and psychological factors.

In clinical trials, placebo-controlled studies are ubiquitous. The placebo effect is characterized by a perceived improvement resulting from the psychological effect of receiving care rather than the actual or no treatment. The success of placebo-controlled trials is primarily due to their double-blind design, which controls patient and assessor expectations, thereby controlling the placebo effect [6].

Clinical trials for chronic thromboembolic pulmonary hypertension (CTEPH) have shown variability in the placebo effect, with some trials reporting significant improvements in exercise capacity and hemodynamic measurements in placebo groups [7,8].

Therefore, we aimed to assess the placebo effect in CTEPH trials evaluating subjective outcomes such as the distance in 6 min walking test (6MWT) and quality of life, as well as other objective measures within the placebo arms of trials. Using CTEPH as a model of disease, we expect that these data could be useful to assess the transversality of the placebo effect, to improve the understanding about the expectations of patients and investigators in CTEPH clinical trials.

## 2. Methods

This systematic review with meta-analysis was conducted following the Preferred Reporting Items for Systematic Reviews and Meta-Analyses (PRISMA) guidelines for healthcare intervention evaluations [9]. The protocol is registered with doi:10.17605/OSF.IO/X35NW.

We included all published randomized placebo-controlled trials (RCTs) evaluating pharmacologic or interventional treatments for chronic thromboembolic pulmonary hypertension (CTEPH). Trials were eligible if they reported pre–post data for six-minute walk test (6MWT) and/or quality of life (QoL) outcomes in placebo arms. Secondary outcomes included hemodynamic parameters, such as mean pulmonary artery pressure (mPAP), pulmonary vascular resistance (PVR), cardiac index, and NT-proBNP levels. We excluded observational studies, case reports, studies without a placebo arm, and perioperative interventions.

A thorough search was performed across MEDLINE and Cochrane Central Register of Controlled Trials (CENTRAL). We examined all the records retrieved from inception until February 2024. Our detailed search methodology is provided in the Table S1 using validated filters [10]. Reference lists were also reviewed for relevant publications. The search was not restricted by language or date.

For study selection, a two-stage review process was employed: Firstly, an independent screening process of titles and abstracts was performed by two reviewers. Subsequently, the full texts of potentially relevant studies were examined for inclusion or exclusion. Any disagreements were discussed until a consensus was reached. The main reason for excluding key studies are depicted in Table S2.

Data were extracted independently by two reviewers using a standardized data extraction form. Extracted variables included trial characteristics, participant demographics, intervention details, and outcome measures. Data were crosschecked for accuracy.

Risk of bias was assessed using the Cochrane Risk of Bias 2.0 tool, which evaluates sequence generation, allocation concealment, blinding, incomplete outcome data, and selective reporting [11]. Each study was rated as having a low, high, or unclear risk of bias. Discrepancies were resolved by consensus.

Meta-analyses were conducted using STATA 17 software. The output included forest plots, showing both individual study outcomes and combined results. In the meta-analysis, we included continuous outcomes, reporting effect sizes as mean differences between baseline and follow-up for each trial arm, along with their corresponding 95% confidence intervals (CIs). We did not calculate standardized effect sizes (e.g., Cohen’s *d*), as our goal was to synthesize absolute changes rather than relative ones. A random-effects model was employed to account for heterogeneity across studies [12]. Heterogeneity was quantified using the I^2^ percentage [13], with values of >50% indicating substantial heterogeneity. Sensitivity analyses and meta-regressions were performed to assess the impact of potential confounders, including baseline characteristics and study duration.

As this study involved secondary analysis of meta-data of previously published trials, ethical approval was not required. The data used were extracted from publicly available sources.

## 3. Results

**Figure 1:**
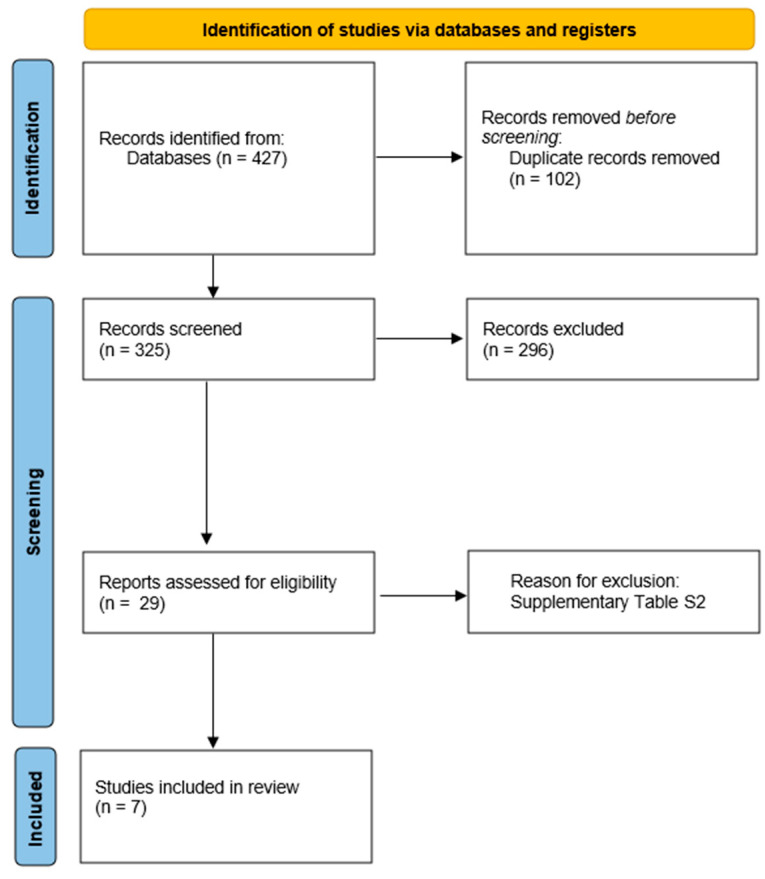
Flowchart of studies selection.

The search and evaluation of studies provided seven placebo-controlled trials with eligible data about 270 CTEPH patients allocated to the placebo arms (Figure 1) [7,8,14,15,16,17,18]. For the MERIT-1, we used the corrected and republished data [8].

A total of seven randomized placebo-controlled trials were included in this systematic review [7,8,14,15,16,17,18], comprising 279 patients allocated to placebo arms. The mean age across studies was 57 years. The median follow-up duration was 16 weeks (range: 12–24 weeks) (Table 1). The risk of bias was low in all randomized controlled trials due to their blinding methods that were appropriate, as well as other characteristics such as absence of significant missing data, adequate measurement of outcomes, and no selective reporting bias (Figure S1).

**Figure 2:**
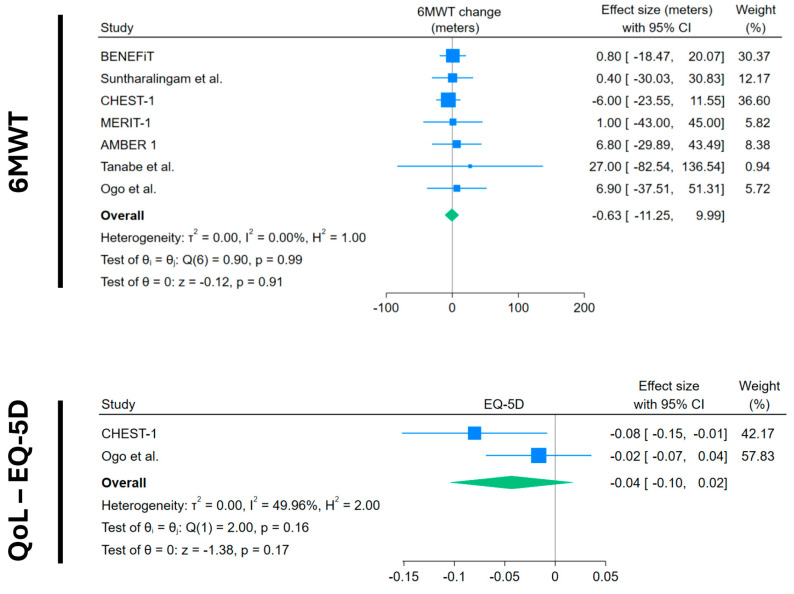
Forest plots of 6MWT and quality of life change in placebo arms. Blue squares with lines represent individual study effect sizes with 95% confidence intervals; square size reflects study weight. The green diamond indicates the pooled effect size and its 95% confidence interval. Studies included with their references: BENEFiT [14]; Suntharalingam et al. [15]; CHEST-1 [7]; MERIT-1 [8]; AMBER 1 [16]; Tanabe et al. [17]; Ogo et al. [18].

**Figure 3:**
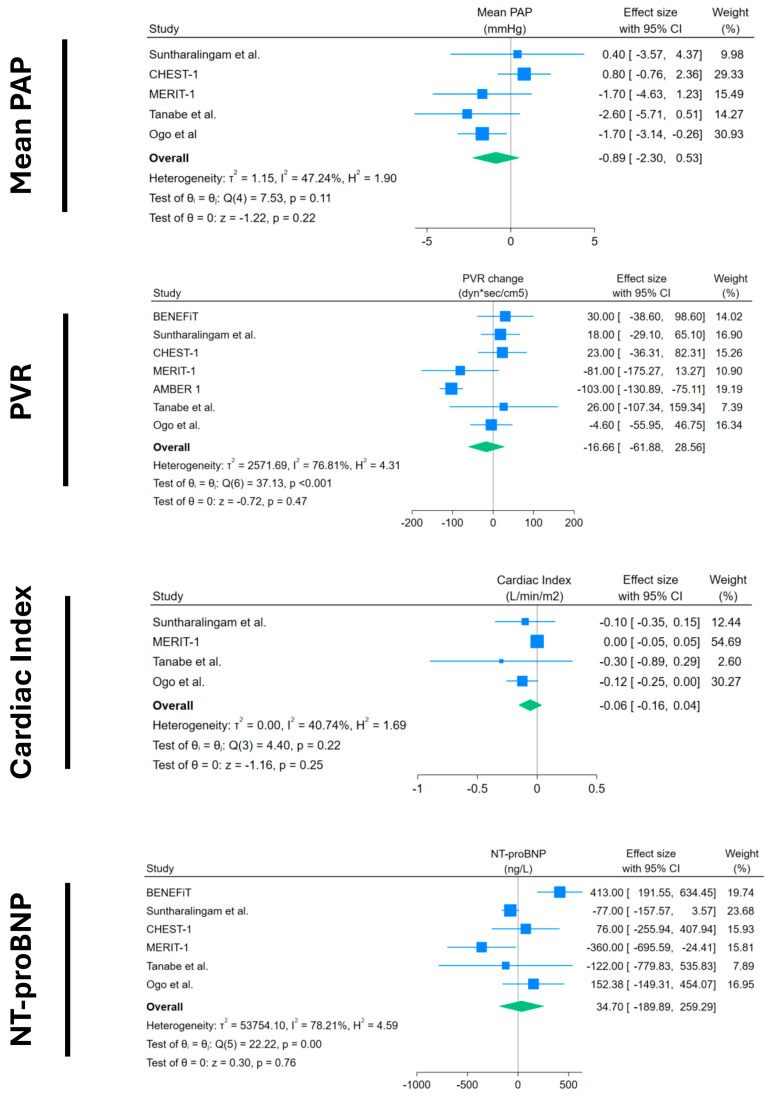
Forest plots of mPAP, PVR, cardiac index, and NT-proBNP change in placebo arms. Blue squares with lines represent individual study effect sizes with 95% confidence intervals; square size reflects study weight. The green diamond indicates the pooled effect size and its 95% confidence interval. Studies included with their references: BENEFiT [14]; Suntharalingam et al. [15]; CHEST-1 [7]; MERIT-1 [8]; AMBER 1 [16]; Tanabe et al. [17]; Ogo et al. [18].

Regarding exercise capacity, seven trials with data from 270 CTEPH patients showed that average 6MWT change in the placebo arm was not significant: –1.31 m (95%CI −12.49 to +9.79) (Figure 2). The quality of life measured through EQ-5D (range of values between −1 and +1) in two trials with 126 patients showed a non-significant decrease in quality of life. (−0.04; 95%CI −0.10 to +0.02) (Figure 3).

Other objective outcomes did not show any significant results: mPAP and PVR showed a non-significant decrease, with a very small magnitude of −0.89 mmHg (95%CI −2.3 to 0.53) and −16.66 dyn × s × cm^−5^ (95%CI −61.88 to +28.56), respectively. Cardiac index and NT-proBNP also showed non-significant changes, with −0.06 L/min/m^2^ (95%CI −0.16 to +0.04) and +34 ng/L (95%CI −189.9 to +259), respectively.

Publication bias analysis was not significant in the Egger test and funnel plot appreciation (Figure S2; *p*-value in Egger test = 0.44).

Sensitivity analyses and meta-regressions (for age, gender, and follow-up time) regarding the results of 6MWT did not change the results (Figures S3–S6; Table S3).

## 4. Discussion

This systematic review with meta-analysis provides a comprehensive assessment of the placebo effect in RCTs involving patients with CTEPH. The primary finding of our analysis suggests that the placebo effect in CTEPH trials is minimal, as demonstrated by the negligible changes in the 6MWT distance, quality of life, and key hemodynamic parameters. Taking the 6MWT as an example, the average decrease of 0.63 m in this evaluation, beyond negative, is far from the landmark 33 m improvement used to define the minimum important clinical difference in PAH patients [19]. These findings hold important implications for the design and interpretation of clinical trials in this population.

The role of placebo in pulmonary hypertension trials has been previously explored in studies evaluating pulmonary arterial hypertension (PAH), which shares pathophysiological similarities with CTEPH. However, PAH is primarily a disease of the small pulmonary arteries, characterized by vasculopathy, endothelial dysfunction, and vascular remodeling, whereas CTEPH is predominantly a mechanical thrombotic obstructive disorder. These distinctions influence treatment approaches, with PAH typically managed using targeted vasodilatory therapies, while CTEPH may require additional surgical intervention or interventional procedures, such as pulmonary endarterectomy (PEA) or balloon pulmonary angioplasty (BPA).

A systematic review assessing the short-term impact of placebo therapy in PAH demonstrated that placebo-treated patients frequently experience clinical deterioration, including worsening of 6MWT and pulmonary hemodynamics [20]. This contrasts with our findings, where no significant deterioration or improvement was observed in the placebo arms of CTEPH trials. One possible explanation for this discrepancy is the inherent difference in disease pathophysiology, with CTEPH being predominantly a mechanical obstruction disorder rather than a primary vasculopathy.

A key consideration in interpreting our findings is the potential for regression to the mean, a statistical phenomenon where extreme baseline values tend to shift toward the average over time. A recent analysis of PAH trials found that placebo effects in 6MWT are negligible when RTM is accounted for [21]. This aligns with our results, reinforcing the notion that the observed changes in placebo groups of CTEPH trials are unlikely to be clinically meaningful. However, it is important to recognize that certain factors, such as patient expectations, trial design, and duration, may still influence placebo responses.

The findings from our study also align with those of previous reviews on the evolution of pharmacological interventions in CTEPH. The introduction of riociguat, the only currently approved pharmacologic therapy for CTEPH, was supported by the CHEST-1 trial, which demonstrated significant improvements in 6MWT and pulmonary hemodynamics compared with placebo [22]. Notably, even in this well-conducted trial, the placebo group did not exhibit any substantial improvement in functional capacity, further supporting our conclusion that placebo responses in CTEPH were not statistically significant, and their magnitude was small. This suggests that the placebo/sham effect varies across conditions [4,5], and that in CTEPH, the physiological reserve might not be available due to the severity and limiting nature of the condition, as well as its evolutive nature.

We would like to emphasize that our data should not discourage the placebo-controlled trials despite the overall small magnitude of placebo effects in the setting of pulmonary hypertension/CTEPH, as there are still many areas where they can be applied [23]. Actually, the ethical considerations surrounding the use of placebo in CTEPH trials are already being considered. Given the progressive nature of CTEPH, withholding active therapy in clinical trials could lead to a worsening of symptoms. The most common trial designs are active-controlled or placebo-controlled trials with novel treatments being tested as an add-on to standard therapy [21]. Our data also suggest that future trials should account for the lack of clinically important placebo effect in the stopping rules of placebo-controlled trials to minimize patient exposure to placebo.

Recent advances in medical and interventional treatments for venous thromboembolic disease, along with their increasing implementation [24,25,26], have raised expectations that the overall burden of CTEPH may decline. In particular, we anticipate that milder forms of CTEPH and/or recurrences following pulmonary vascular interventions may be associated with a more pronounced placebo effect than what was observed in the trials included in this systematic review and meta-analysis. Therefore, we underscore that the placebo effect observed in current trials may not be directly comparable to that in future CTEPH studies.

The limitations of our analysis include the relatively small sample size of included placebo arms and heterogeneity in trial designs. Moreover, while we focused on pre–post changes within placebo groups, we did not compare these findings against active treatment arms in detail. Future studies should aim to expand the evidence base by incorporating larger datasets and exploring additional patient-centered outcomes.

## 5. Conclusions

The placebo effect in CTEPH trials was not statistically significant and had a modest magnitude. Our results are informative for investigators and patients and despite this absence of relevant improvements in the placebo arms, it should not discourage the use of placebo-controlled trials where applicable and ethical.

## Figures and Tables

**Table 1:** Overall characteristics of included studies.

Trial	N Placebo Arm	Age (Mean/SD or Median/IQR)	% Females	BMI	Background PH Therapy	PEA/BPA	Follow-Up
**BENEFiT [14]**	80	63.1 ± 10.3	58.8	N/A	N/A	PEA 27.5%	16 weeks
**Suntharalingam et al. [15]**	10	60.0 ± 14.4	30	29.8 ± 6.1	N/A	PEA 70%	12 weeks
**CHEST-1 [7]**	88	59 ± 13	61	28 ±5	N/A	PEA 23%	16 weeks
**MERIT-1 [8]**	40	56.9 ± 13.9	63	26.2 ± 4.0	63%	N/A	24 weeks
**AMBER 1 [16]**	13	59.8 ± 9	63	27.1 ± 6.8	0%	0%	16 weeks
**Tanabe et al. [17]**	9	60 ± 5	77.8	N/A	55.6%	PEA 11.1%	17 weeks
**Ogo et al. [18]**	39	68.3 ± 9.6	74.4	N/A	66.7%	PEA 12.8% BPA 56.4%	20 weeks

BMI: body mass index; BPA: balloon pulmonary angioplasty; N/A: not available; PEA: pulmonary endarterectomy; PH: pulmonary hypertension; SD: standard deviation. Studies included with their references: BENEFiT [14]; Suntharalingam et al. [15]; CHEST-1 [7]; MERIT-1 [8]; AMBER 1 [16]; Tanabe et al. [17]; Ogo et al. [18].

## Supplementary Materials

The following supporting information can be downloaded at: https://www.mdpi.com/article/10.3390/medsci13020057/s1, Table S1: Search strings for OVID (MEDLINE and CENTRAL); Table S2: Studies excluded in full-text decision stage and reasons; Table S3: Results of univariate meta-regression regarding 6MWT. Figure S1: Risk of bias using RoB 2.0 for the outcome 6MWT; Figure S2: Funnel plot and Egger test result (*p*-value 0.44); Figure S3: Jackknife leave-one-out sensitivity analysis; Figure S4: Bubble plot of meta-regression of age impact in 6MWT in placebo arms; Figure S5: Bubble plot of meta-regression of % of female patients impact in 6MWT in placebo arms; Figure S6: Bubble plot of meta-regression of follow-up time impact in 6MWT in placebo arms.
