# Supplementary material for: The Placebo Effect in Chronic Thromboembolic Pulmonary Hypertension Trials: A Systematic Review and Meta-Analysis
Source: Med Sci (Basel). 2025 May 7;13(2):57. doi: 10.3390/medsci13020057 (PMC12101364; doi:10.3390/medsci13020057)
Supplement: Supplementary file 1 [file medsci-13-00057-s001.zip › medsci-3516392-Supplementary.pdf]

Supplementary Data

The Placebo Effect in Chronic Thromboembolic Pulmonary Hypertension Trials: A Systematic Review and Meta-Analysis

Daniel Caldeira, Daniel Inácio Cazeiro, Rui Plácido, Filipa Ferreira, Rita Calé and Fausto J. Pinto

Published in Medical Sciences

Table S1: Search strings for OVID (MEDLINE and CENTRAL)

#	Searches
1	chronic thromboembolic pulmonary hypertension.af.
2	cteph.ti,ab.
3	chronic pulmonary embolism.ti,ab.
4	1 or 2 or 3
5	sham.af.
6	placebo.af.
7	random*.ti,ab.
8	5 or 6 or 7
9	4 and 8

Table S2: Studies excluded in full-text decision stage and reasons.

Keys studies excluded	Reasons for exclusion
**Ulrich 2017**	Cross-over trial without data for CTEPH and without pre-post data in control arm
**PEA bridging study**	Trial registry - only with congress communication; https://www.cteph-association.org/pea-bridging-study.html
**Ulrich 2017**	Duplicated
**Ghofrani H 2017**	Congress presentation of MERIT; Duplicated
**Jansa 2013**	Duplicated data from CHEST-1
**Gofrani 2013**	Duplicated
**Zeng 2012**	No specific data for CTEPH
**Benza 2012**	Evaluation of REVEAL score in CTEPH patients of CHEST
**Olschewski 2002**	No specific data for CTEPH
**Lichtblau 2023**	No specific data for CTEPH and cross-over design
**Milne 2023**	no CTEPH
**Kim 2023**	Congress presentation of SELECT
**Jenkins 2022**	Congress presentation
**Muller 2023**	Congress presentation of Lichtblau
**Lichtblau 2021**	Acute intervention and cross-over
**Boutou 2021**	No specific data for CTEPH
**Schneider 2020**	Cross-over trial without data for CTEPH and without pre-post data in control arm
**Ulrich 2019**	No specific data for CTEPH
**Romanov 2020**	active control
**Kramm 2005**	Perioperative acute intervention
**Reesink 2010**	no placebo
**Sadushi-Kolici 2019**	no placebo

Table S3: Results of univariate meta-regression regarding 6MWT

Meta-regression	Meters	95%CI	p-value
**Age (per year)**	0.916	-3.08 to 4.91	0.65
**Female (%)**	0.055	-0.91 to 1.02	0.91
**Follow-up (weeks)**	0.342	-3.81 to 4.49	0.87

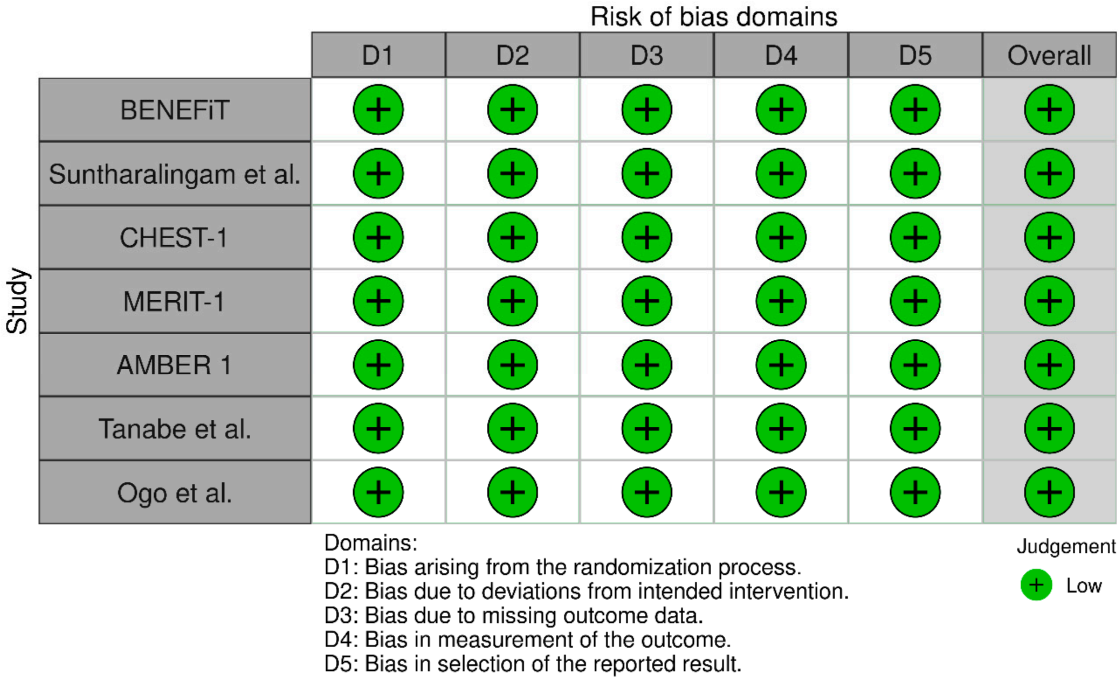

Figure S1: Risk of bias using RoB 2.0 for the outcome 6MWT.

.

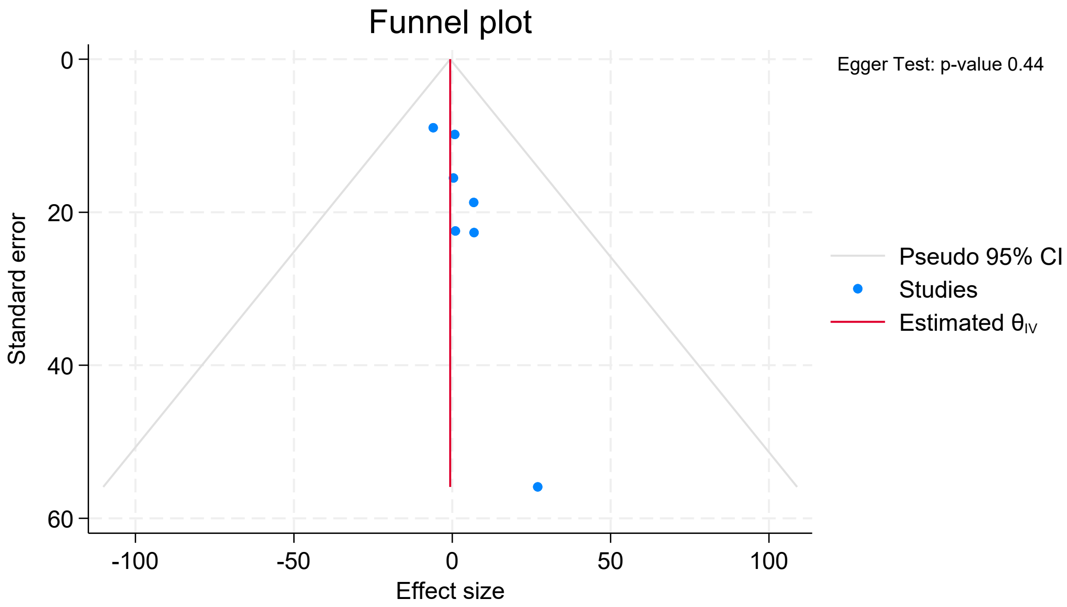

Figure S2: Funnel plot and Egger test result (p-value 0.44)

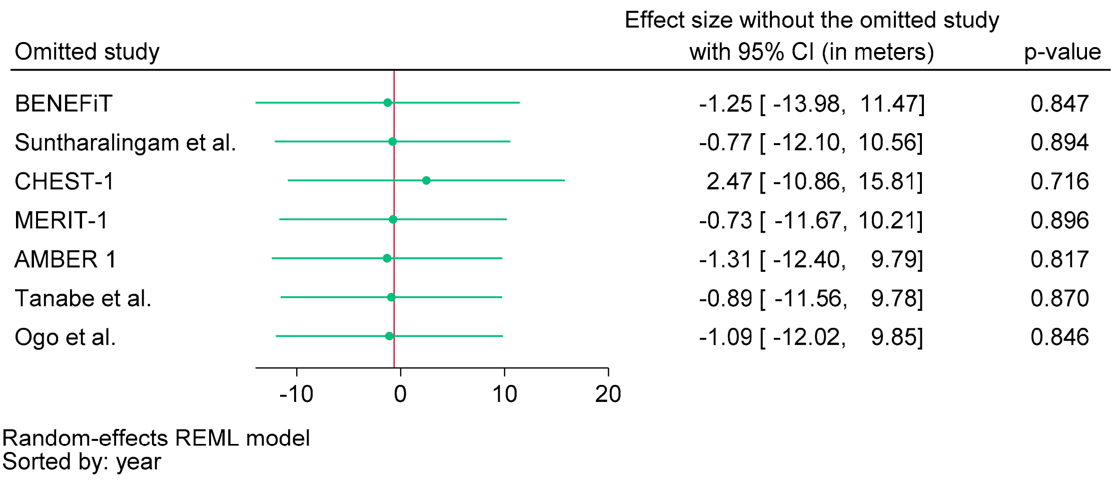

Figure S3: Jackknife leave-one-out sensitivity analysis

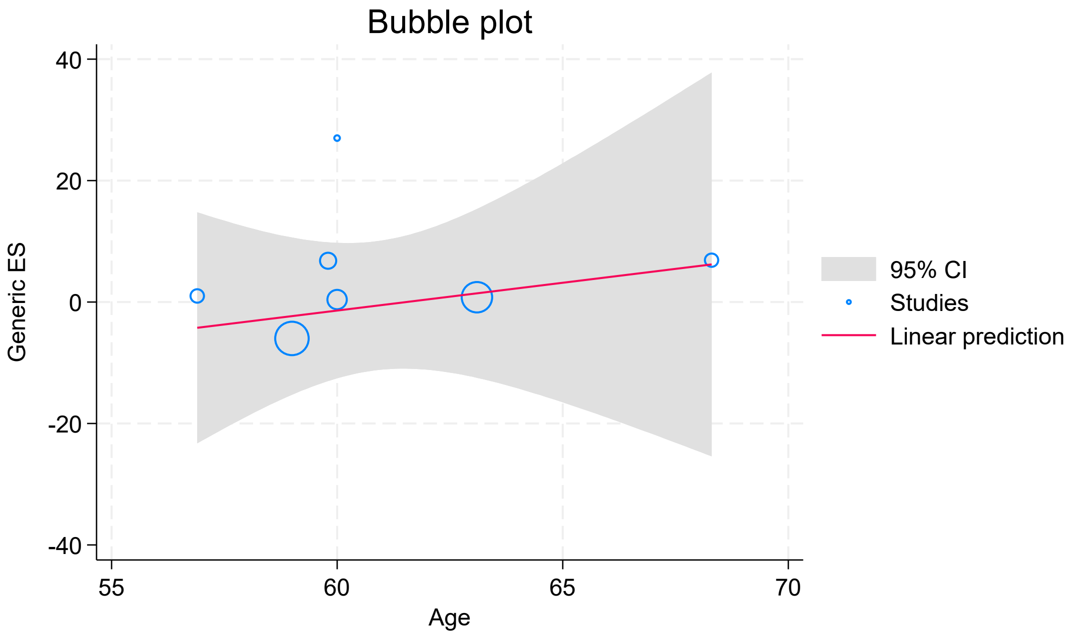

Figure S4: Bubble plot of meta-regression of age impact in 6MWT in placebo arms.

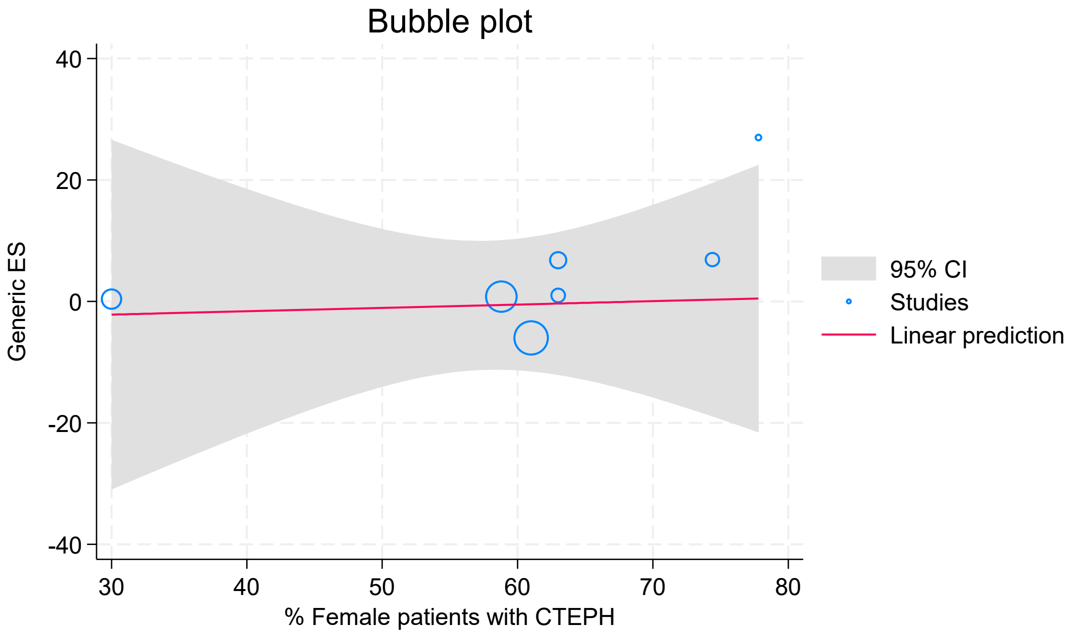

Figure S5: Bubble plot of meta-regression of % of female patients impact in 6MWT in placebo arms.

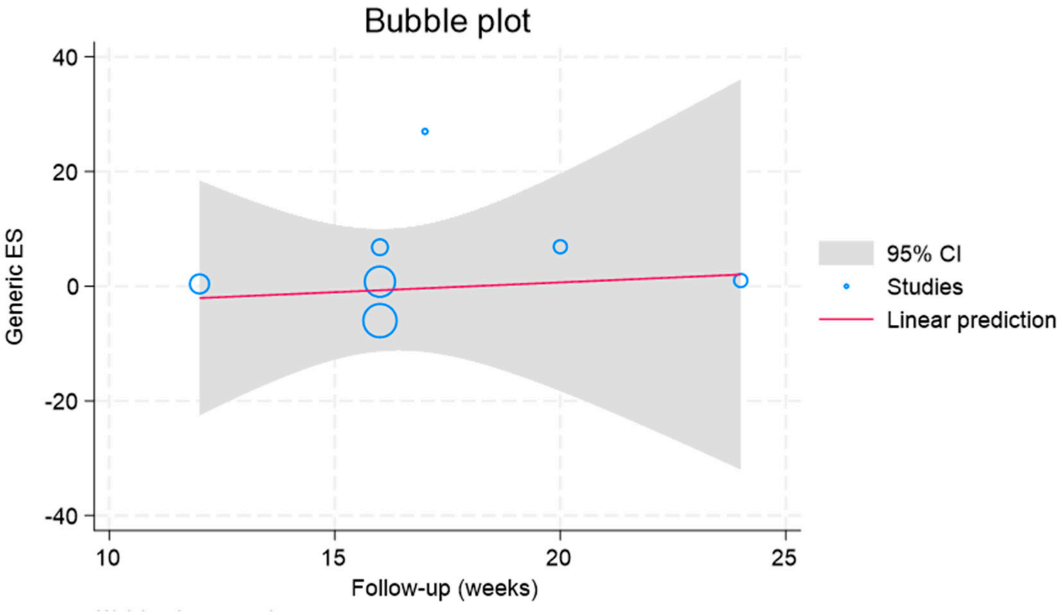

Figure S6: Bubble plot of meta-regression of follow-up time impact in 6MWT in placebo arms.
